# Bibliometric and visual analysis in the field of two-dimensions nano black phosphorus in cancer from 2015 to 2023

**DOI:** 10.1007/s12672-024-01104-y

**Published:** 2024-07-03

**Authors:** Jing’an Huang, Ling Zhang, Boren Li, Yuanchu Lian, Xiaoxin Lin, Zonghuai Li, Bo Zhang, Zhongwen Feng

**Affiliations:** 1 Scientific Research Center, Guilin Medical University, Guilin, China; 2 Department of Pharmacy, Guangxi Academy of Medical Sciences and the People’s Hospital of Guangxi Zhuang Autonomous Region, Nanning, China

**Keywords:** Bibliometric analysis, Nano black phosphorus, Photothermal therapy, Drug delivery, Immunotherapy

## Abstract

This study aims to provide a comprehensive summary of the status and trends of Two-Dimensional Nano Black Phosphorus (2D nano BP) in cancer research from 2015 to 2023, offering insights for future studies. To achieve this, articles from the Web of Science database published between 2015 and 2023 were analyzed using R and VOSviewer software. The analysis included 446 articles, revealing a consistent increase in publication rates, especially between 2017 and 2019. China emerged as a leader in both publication volume and international collaborations. Prominent journals in this field included ACS Applied Materials & Interfaces and Advanced Materials, while key researchers were identified as Zhang Han, Tao Wei, and Yu Xuefeng. The analysis highlighted common keywords such as drug delivery, photothermal therapy, photodynamic therapy, and immunotherapy, indicating the major research focuses. The findings suggest that 2D nano BP holds significant promise in cancer treatment research, with a growing global interest. This study thus serves as a valuable reference for future investigations, providing a detailed analysis of the current state and emerging trends in this promising field.

## Introduction

Cancer is a chronic disease that has a significant impact on individuals' health, drastically reducing their quality of life and often resulting in death [1]. In 2020 alone, it was estimated that nearly 19.3 million new cases of cancer have been reported globally, with a staggering worldwide death toll of 10 million. Projections indicate that by the year 2040, the number of cancer patients will increase to 28.4 million globally, by calculating in advance, signifying a 47% increase compared to 2020 [2]. These statistics highlight the urgent need for ongoing research and development in the field of cancer treatment and prevention.

At present, the main methods of cancer treatment are traditional treatments such as surgical therapy [3], radiotherapy [4], and chemotherapy [5]. In the comparison between the previous three, chemotherapy and adjuvant therapy have been widely used in clinical practice for the treatment of cancer syndromes due to their excellent inhibitory effect on the spread and metastasis of cancer cells [6]. However, traditional chemotherapy also has certain drawbacks in terms of therapeutic efficacy, such as: large cytotoxicity and, unclear therapeutic targets, and painful treatment process [7].

In comparison, emerging cancer treatment methods such as targeted therapy [8], photodynamic therapy(PDT), photothermal therapy(PTT) [9], and immunotherapy [10] offer several advantages. These methods exhibit lower toxicity to the body, more precise targeting of cancer cells, and reduced pain for patients during treatment [11–13]. However, in these emerging cancer therapy, it has been found that the currently used target vectors also have certain limitations. For instance, currently used target vectors have limited specific surface areas, often requiring multiple treatment sessions [14]; Pharmacokinetic distribution is deficient and cannot cross specific biological barriers (blood–brain barrier) to treat the brain cancer [15].

As PTT and PDT are increasingly utilized in clinical treatments for various types of cancer, their limitations are gradually becoming apparent. In PDT and PTT, near-infrared (NIR) light is frequently selected as the illumination source, with wavelengths typically spanning from 700 nm to 2500 nm [16]. The choice of NIR is strategic, leveraging its superior tissue penetration capabilities to augment both the depth and efficacy of the treatment, thereby facilitating access to deeper tissue layers. However, the penetration depth of light is limited by tissue absorption and scattering, thus restricting its therapeutic depth [17]. Potential toxicity: Currently, the available types of photosensitizers are limited, and most of them belong to nano inorganic materials [18]. With the increase of treatment times, if these materials cannot be metabolized and excreted by the human body in a timely manner, they will accumulate in the body, the potential toxicity of cells is a concern [19]. Photosensitizer instability: Many photosensitizers have been found to be insensitive to light or susceptible to photodegradation, which affects the therapeutic effect [20].

Immunotherapy holds great promise in cancer treatment, however, it is essential to consider certain limitations. The difficulty of regulating immune adjuvants: selecting appropriate immune adjuvants is crucial for the success of immunotherapy, as immune adjuvants can regulate the activity and degree of immune response of the immune system. However, there is still a lack of ideal materials and methods to accurately control and adjust their sensitivity to the immune system [21]. The enhancement effect of immunotherapy is not significant: Although immunotherapy has shown significant effects in some patients, there are still many patients who are not sensitive or have limited responses to immunotherapy, which may be related to the limited selection of immune adjuvants and the different adaptability of different individuals' immune systems to different immune adjuvants [22]. Currently, focusing on new 2D nanomaterials may offer potential solutions to improve the limitations of targeted therapy, PTT, and immunotherapy [23]. For example, gold nanoparticles have been extensively utilized for various applications, particularly in the treatment of malignant tumors. These applications include drug and nucleic acid delivery, photodynamic therapy, and photothermal therapy [24, 25]. In addition, copper and copper oxide nanoparticles show significant potential as anti-cancer agents, drug delivery systems, and photodynamic therapy enhancers, among other applications [26]. In terms of adjuvant therapy, tantalum oxide nanoparticles are considered one of the most promising contrast agents for computed tomography due to their high X-ray attenuation coefficient, excellent biocompatibility, and easily modified surface chemistry [27]. Among many nanoparticles, BP has gradually attracted the attention of researchers as a new material.

With the deepening of research on 2D nano BP in recent years, researchers were surprised to find that 2D nano BP has unique advantages in the field of drug targeting vectors compared with previous targeting vectors, such as: owing a larger surface area of the same quality, allowing for the delivery of a higher drug load at one time [28]; Better biocompatibility, resulting in fewer toxic side effects on the human body [29], enabling it to overcome biological barriers like the blood–brain barrier and deliver drugs more accurately, controlling the dosage and rate of drug release. [30]. Surprisingly, 2D nano BP also demonstrates great potential in the field of PTT and PDT: It has a high efficiency in converting the light energy contained in light into thermal energy [31], especially the absorption effect of near-infrared light is significant [32], therefore, it is possible to generate adequate heat under low light power, so as to break the limitation of deep tissue therapy; It can improve its stability the targeting of photosensitizers through surface modification or functionalization, and it can adjust the photothermal and photodynamic properties so that the treatment range can be extended to deep tissues to meet the needs of different diseases [33, 34]. In addition, 2D nano BP plays a crucial role in the field of immunotherapy. It can be used as a new immune adjuvant, a substance that can enhance the expression of the immune response, because it has a large specific surface area [35], increasing the surface area in contact with immune cells, to facilitate the delivery of drugs in immunotherapy and improve the effect of immune stimulation [36]. Therefore, owing to its multifaceted functions in nano targeting carriers, PTT, PDT, and immunotherapy, 2D nano BP is expected to become an indispensable tool in the future development of nanomedicine.

The adoption of bibliometric methods in various disciplines has proven to be valuable in organizing and analyzing published literature [37]. Researchers can utilize these methods to gain insights into research trends, cutting-edge topics, and popular directions within specific fields [38]. Through bibliometrics, researchers can conduct quantitative analysis of a large amount of literature data, revealing the cooperative relationships about authors, institutions, and countries, as well as the frequency and co-occurrence of various keywords [39]. Such analysis results help to gain a deeper understanding of the development trends and key areas of research, providing theoretical and literature references for future research in this field [40]. At the same time, it also promotes academic exchange and cooperation [41], and plays a positive role in scientific research policy formulation and resource allocation. Unfortunately, there is still a lack of organization and statistics on the research literature published on 2D nano BP in the field of cancer treatment. Although the number of research papers related to 2D nano BP in the field of cancer treatment has steadily increased, the overall development of knowledge, research hotspots, and research trends is still unclear. Thus, in this study, in order to visualize and analyze the data, we applied R software, we used R software, VOSviewer, and CiteSpace to analyze relevant literature on the application of 2D nano BP in the field of cancer treatment. Our goal is to explore the changes and development trends in the research hotspots of 2D nano BP in the field of cancer treatment from 2015 to 2023, and identify potential research hotspots to offer theoretical assistance for future exploration. Looking ahead and having a better understanding of the current situation and potential is crucial for the sustainable development of this field.

## Materials and methods

### Literature sources and retrieval strategies

Database: Web of Science (WOS) database core data collection. (2) Retrieved on July 28, 2023; Search steps: following Table 1; Search period: July 28, 2015 to July 28, 2023; Choose "article" or "review" for the type of literature. (3) Export the literature to plain text format and name it "download_XXX".

**Table 1:** Retrieval steps and results

Steps	Searches	Results
#1	(((((TS = (“Black phosphorus”)) OR TS = (“black phosphorus nanosheets”)) OR TS = (“black phosphorus quantum dots”)) OR TS = ( “Black phosphorus BP”)) OR TS = (“few layer black phosphorus”)) OR TS = (“black Phosphorene”)	7560
#2	(((((((((((((((((TS = (Cancer)) OR TS = (Tumor))) OR TS = (Tumors)) OR TS = (Neoplasm)) OR TS = (Neoplasia)) OR TS = (Neoplasias)) OR TS = (Cancers)) OR TS = (“Malignant Neoplasm”)) OR TS = (Malignancy)) OR TS = (Malignancies)) OR TS = (“Malignant Neoplasms”)) OR TS = (“Neoplasm Malignant”)) OR TS = (“Neoplasms Malignant”)) OR TS = (“Benign Neoplasms”)) OR TS = (“Benign Neoplasm”)) OR TS = (“Neoplasms Benign”)) OR TS = (“Neoplasm Benign”)	2,167,004
#3	(((#1 AND #2) AND DOP = (2015–03-22/2023–07-28)) AND DT = (Article OR Review)) AND LA = (English)	446

### Date analysis

In this study, we utilized the previously established research methodology [42, 43]. Briefly, to analyze the annual publications, Origin 2018 was used. Additionally, the bibliometrix package of R software (version 4.22.17675.0), (http: //www.bibliometrix.org), VOSviewer (version 1.6.18), and CiteSpace (version 6.1.4) were employed to visually analyze data and draw scientific knowledge maps. To ensure data accuracy and reliability, two different authors conducted data extraction and analysis management, respectively. VOSviewer was used to visualize the co-authorship network of countries/institutions/authors, co-citation analysis of sources, and co-occurrence of keywords. In co-authorship network analysis, we set the following parameters: minimum number of documents of a country ≥ 5; minimum number of documents of an organization ≥ 5; minimum number of documents of an author ≥ 5. In the co-citation of source analysis, we set the following parameters: minimum number of citations of a source ≥ 5. Additionally, in the co-occurrence of keyword analysis, the parameters were set as follows: minimum number of occurrences of a keyword ≥ 5, and we excluded “black phosphorus”, “cancer therapy”, “black phosphrous”,“cancer” and “tumor therapy” keywords. The journal impact factors (IFs) were retrieved from Journal Citation Reports (JCR) of 2022.

## Results

### General landscapes of included documents on 2D nano BP in *cancer*

**Fig. 1:**
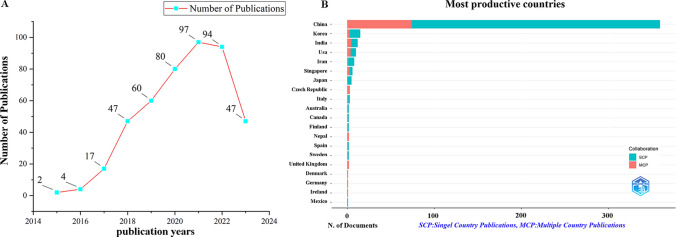
Trends in annual publication outputs in the field of 2D nano BP in cancer from 2015 to 2023. **A** Trends of annual publication outputs. **B** Showcases the distribution of countries and collaborative efforts among corresponding authors

**Fig. 2:**
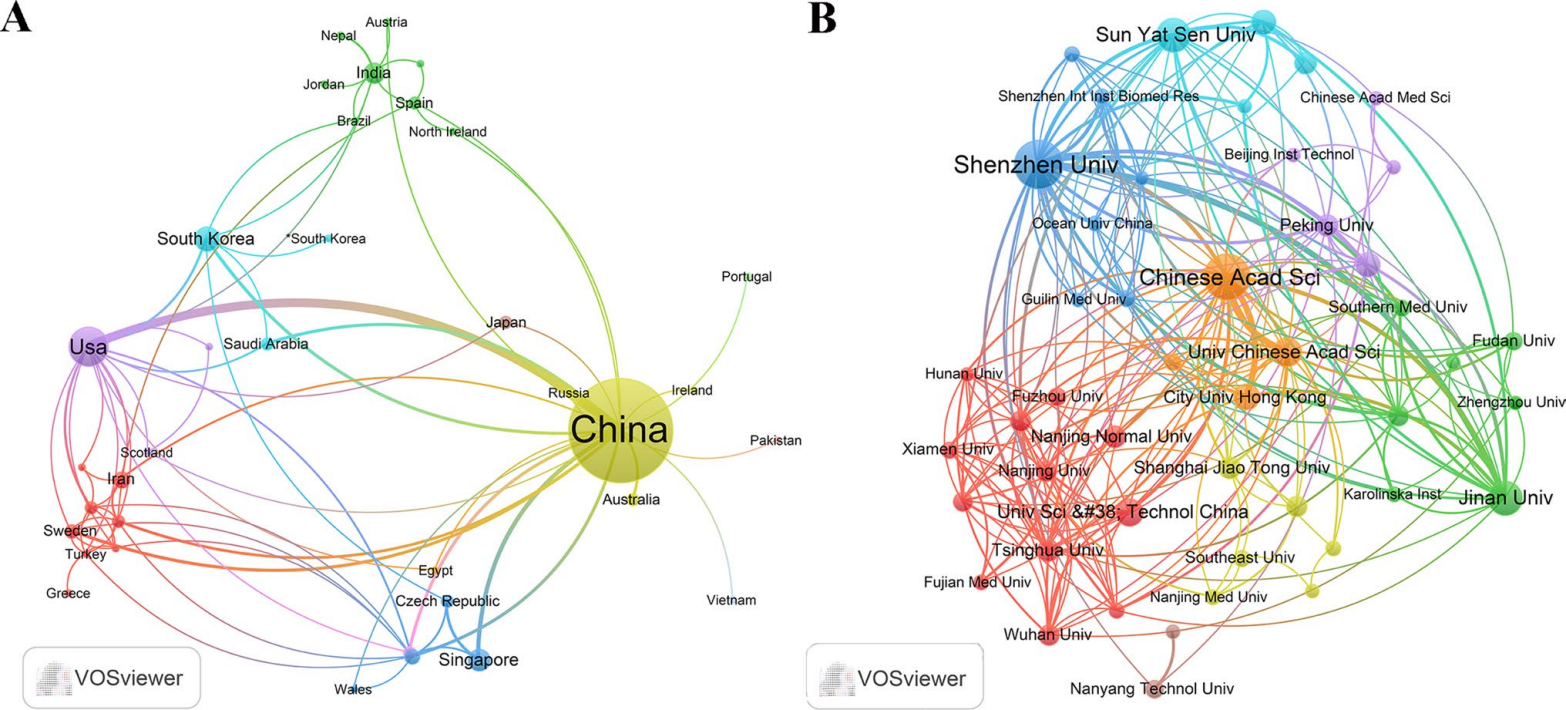
The map of countries and institutions in the field of 2D nano BP in cancer from 2015 to 2023

A total of 446 documents were collected from Web of Science Core Collection(WoSCC) without duplicates (Fig. 1A). The figure illustrates the increasing trend of publications related to 2D nano BP in cancer treatment over the years. Specifically, between 2015 and 2017, with a gradual increase about the quantity of relevant documents. However, between 2018 and 2022, there was a extremity increase amplitude in research output, with 97 papers published in 2021 alone. As of 28 July 2023, an additional 47 relevant documents had been published, further contributing to the growing body of literature in this area. Based on the corresponding authors’ countries, we found that China (n = 359) had been the most productive, followed by Korea (n = 15), India (n = 12), United States (n = 10), and Iran (n = 8). Interestingly, we found the top five countries with the rank of the most publication, only 20.00% and 12.50% from Korea and Iran were multiple country publications (MCPs), respectively, which is well below 41.70% and 40.00% in the India and United States, respectively (Fig. 1B**; **Table 2). Meanwhile, Fig. 2A indicates that China has shown the most extensive collaboration with other countries in the field of 2D nano BP in cancer treatment, Chinese researchers and institutions have actively collaborated with international counterparts to advance knowledge and research in this area. Additionally, the collaboration map demonstrates that Shenzhen University (n = 64) and Chinese Academy of Sciences (n = 58) were representative centers of collaboration **(**Fig. 2B; Table 3**)**.

**Table 2:** Most relevant countries by corresponding authors of 2D nano BP in cancer

Country	Articles	SCP	MCP	Freq(%)	MCP_Ratio(%)
China	359	285	285	80.90%	20.60%
Korea	15	12	12	3.40%	20.00%
India	12	7	7	2.70%	41.70%
Usa	10	6	6	2.30%	40.00%
Iran	8	7	7	1.80%	12.50%
Singapore	6	3	3	1.40%	50.00%
Japan	5	5	5	1.10%	0.00%
Czech Republic	3	0	0	0.70%	100.00%
Italy	3	2	2	0.70%	33.30%
Australia	2	1	1	0.50%	50.00%

*MCP* multiple-country publication, *SCP* single-country publication

**Table 3:** Top 10 most relevant affiliations of 2D nano BP in cancer

Rank	Affiliation	Articles (n)
1	Shenzhen University	64
2	Chinese Academy of Sciences	58
3	Jinan University	31
4	Sun Yat Sen Universicty	31
5	University of Chinese Academy of Sciences	23
6	University of Science and Technology of China	20
7	City University of Hong Kong	19
8	Harvard Medical School	17
9	Central South University	14
10	Tsinghua University	13
10	Peking University	13
10	Shanghai Jiao Tong University	13
10	Nanjing Normal University	13

### Journals and co-cited journals

The Bibliometrix and ggplot2 packages of R software (version 4.22.17675.0) were used to analyze the journals with the most published documents and the journals with the most citations in this field.

**Fig. 3:**
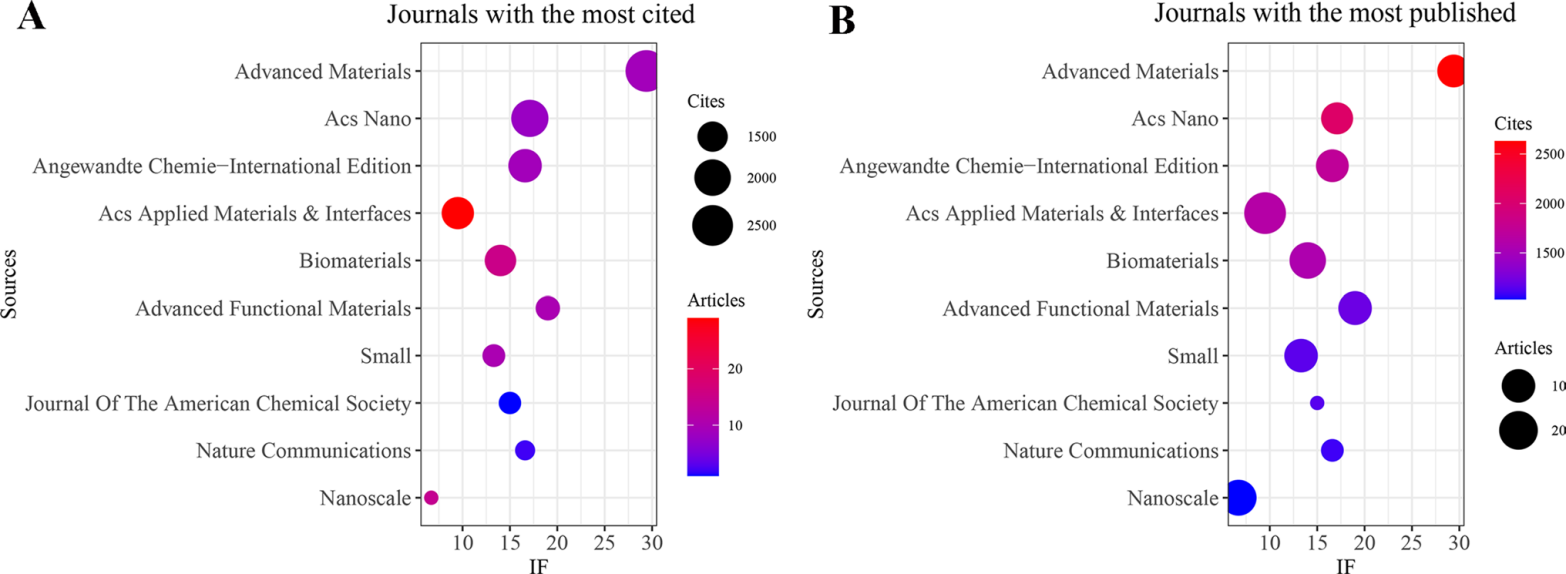
The journal with the largest number of articles published and the journal with the largest number of citations. **A** The journal with the highest count of published documents. **B** The journals with the highest count of citations

**Fig. 4:**
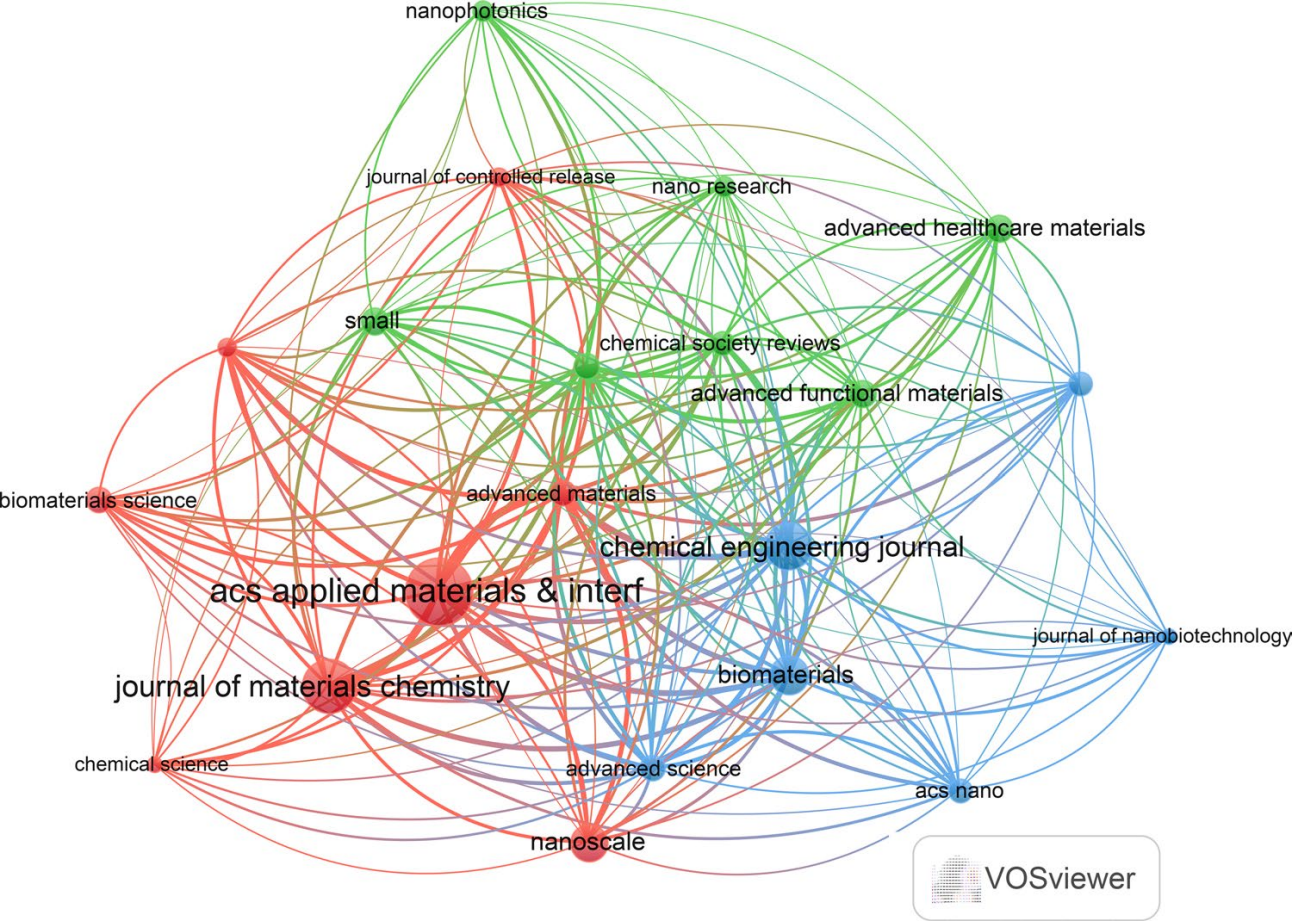
Co-cited journals involved in the field of 2D nano BP in cancer

Additionally, VOSviewer (version 1.6.18) was utilized to conduct the co-cited journal analysis. The results indicated that 446 documents were published in 153 academic journals **(**Table 4**)**. Figure 3A show that the most frequently cited journals were the Advanced Materials (n = 2,629, IF = 29.4), followed by Acs Nano (n = 2,070, IF = 17.1), Angewandte Chemie-International Edition (n = 1,723 IF = 16.6), Acs Applied Materials & Interfaces (n = 1,631, IF = 9.5), and Biomaterials (n = 1,573, IF = 14). Moreover, Table 5; Fig. 3B demonstrate that the most published documents were in the Journal of Acs Applied Materials & Interfaces (n = 29, IF = 9.5), followed by Materials Chemistry B (n = 22, IF = 7), Chemical Engineering Journal (n = 19, IF = 15.1), Nanoscale (n = 14, IF = 6.7), and Advanced Functional Materials (n = 10 IF = 19). The co-cited journal maps revealed that the Journal of Ethnopharmacology and Evidence-Based Complementary and Alternative Medicine were representative centers of collaboration **(**Fig. 4**)**. These findings suggest that the Journal of Acs Applied Materials & Interfaces and Journal of Materials Chemistry B and Chemical Engineering Journal may be influential journals in the field of 2D nano BP in cancer. Furthermore, these data indicate that there is still a lack of publications on the outcomes of 2D nano BP in cancer in high-impact journals. This further indicates the need to improve the depth and quality of research in this field.

**Table 4:** Top 10 journals with the most cited journals

Journal	Cites	Articles	IF(2022)
Advanced Materials	2629	9	29.4
Acs Nano	2070	8	17.1
Angewandte Chemie-International Edition	1723	9	16.6
Acs Applied Materials & Interfaces	1631	29	9.5
Biomaterials	1573	15	14
Advanced Functional Materials	1208	10	19
Small	1157	10	13.3
Journal Of The American Chemical Society	1146	1	15
Nature Communications	1090	2	16.6
Nanoscale	1035	14	6.7

**Table 5:** Top 10 journals with the most published articles

Journal	Articles	Cites	IF(2022)
Acs Applied Materials & Interfaces	29	1631	9.5
Journal Of Materials Chemistry B	22	598	7
Chemical Engineering Journal	19	369	15.1
Biomaterials	15	1573	14
Nanoscale	14	1035	6.7
Advanced Functional Materials	10	1208	19
Advanced Healthcare Materials	10	348	10
Small	10	1157	13.3
Advanced Materials	9	2629	29.4
Advanced Science	9	542	15.1

### Authors and co-cited authors

**Fig. 5:**
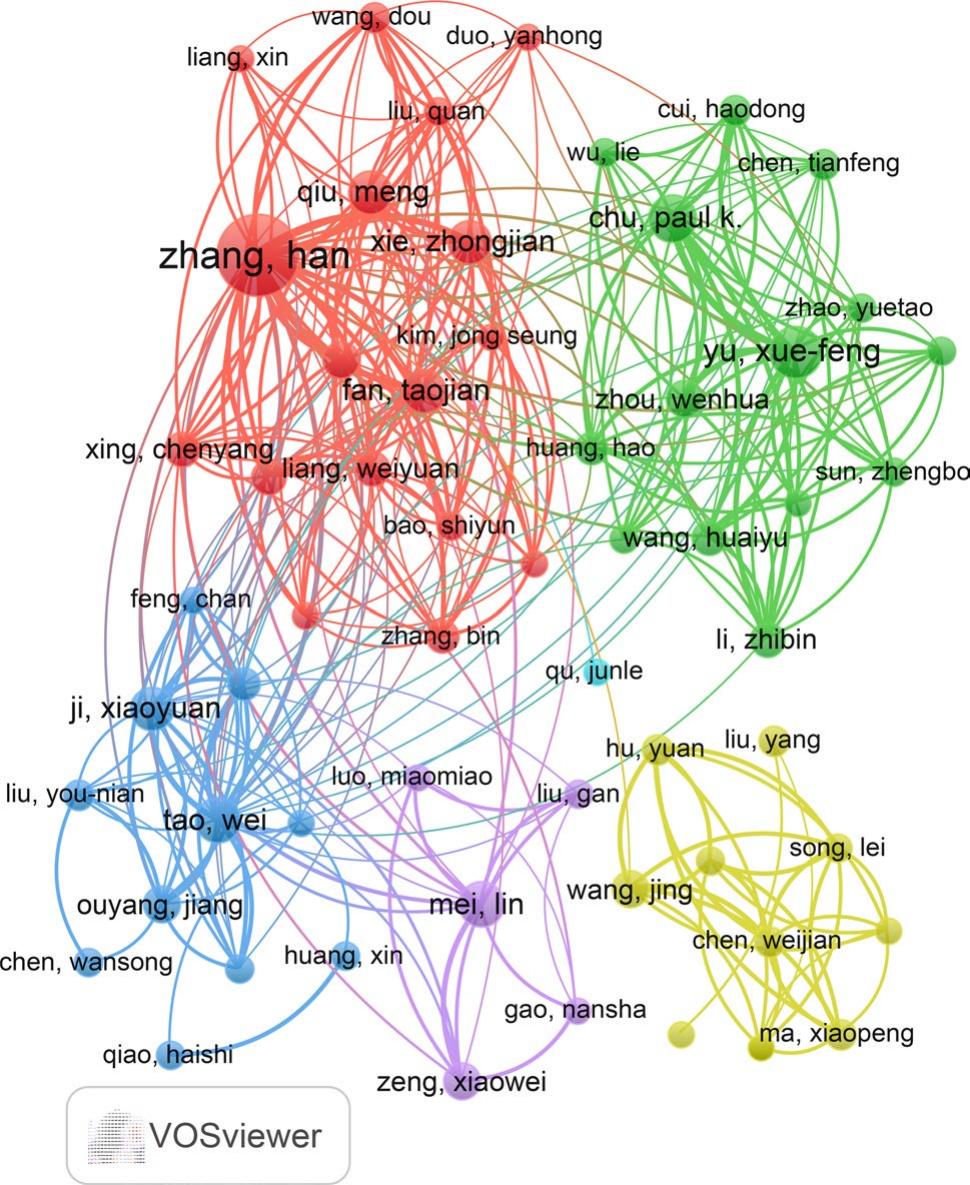
The map of co-authorship in the field of 2D nano BP in cancer from 2015 to 2023

The Table 6 presents the top ten authors based on the quantity of published papers. Among these, the top five are Zhang Han (n = 51), Yu Xuefeng (n = 20), Chu Paul K (n = 17), Tao Wei (n = 16), and Mei Lin (n = 16). In Table 7, we outline the top ten authors with the highest number of citations in the literature. The leading five authors in terms of citations are Zhang Han (n = 6873), Yu Xuefeng (n = 2857), Tao Wei (n = 2772), Chu Paul K (n = 2746), and Wang Huaiyu (n = 2345). The number of publications reflects the authors' research productivity to some extent, while the number of citations in the literature indicates their influence. Notably, Zhang Han, Yu Xuefeng, Chu Paul K, and Tao Wei simultaneously rank as the top five authors in both the number of publications and the number of citations. This underscores their significant contributions to the field and their status as the most influential authors within it. It is noteworthy that the generality of these authors are from China, highlighting the considerable importance and influence of Chinese researchers in this field. Furthermore, our network diagram of co-authors (Fig. 5**)** reveals extensive and close collaboration among authors, with Zhang Han, Tao Wei, and Yu Xuefeng serving as representative centers of collaboration. This finding underscores their profound impact on the field.

**Table 6:** Top 10 Documents authors related to 2D nano BP on cancer

Rank	Author	Documents	Citations
1	Zhang Han	51	6873
2	Yu Xuefeng	20	2857
3	Chu Paul k	17	2746
4	Tao,Wei	16	2772
5	Mei Lin	16	2227
6	Xie Zhongjian	15	1359
7	Fan Taojian	15	862
8	Qiu Meng	14	1912
9	Ji Xiaoyuan	14	2462
10	Li Zhongjun	11	1961

**Table 7:** Top 10 citations authors related to 2D nano BP on cancer

Rank	Author	Citations	Documents
1	Zhang Han	6873	51
2	Yu Xuefeng	2857	20
3	Tao Wei	2772	16
4	Chu Paul K	2746	17
5	Ji Xiaoyuan	2462	14
6	Wang Huaiyu	2345	9
7	Mei Lin	2227	16
8	Shao Jundong	2052	6
9	Xie Hanhan	1967	5
10	Li Zhongjun	1961	11

### References analysis

**Fig. 6:**
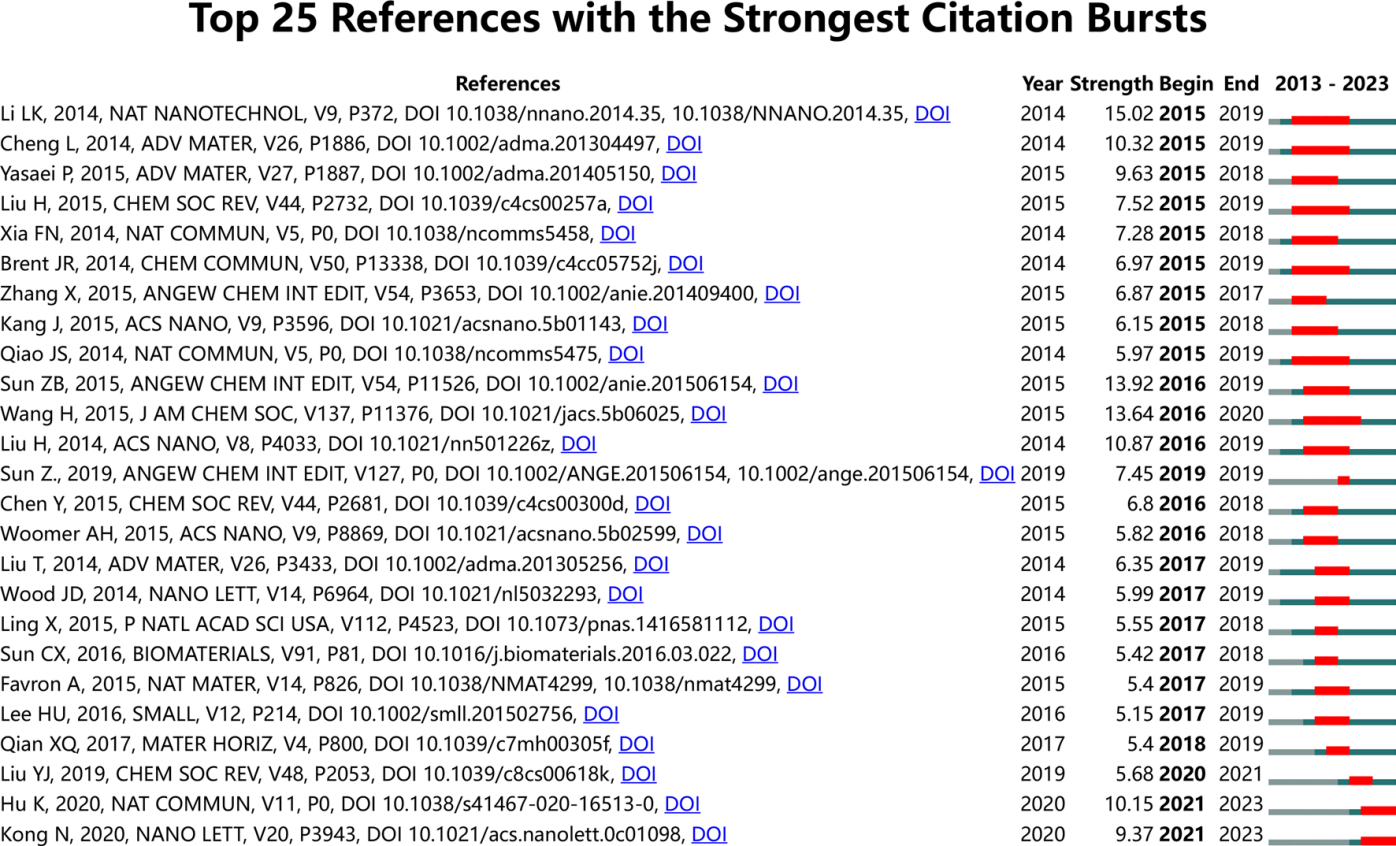
Top 25 References with the strongest citation bursts on 2D nano BP in cancer

We utilized the bibliometrix package in R software to determine the 25 most frequently cited references within the domain of 2D nano BP and cancer (see Table 8). Our analysis revealed that these references had accrued over 200 citations and originated from 15 distinct journals, underscoring the necessity for continued investigation in this area. Notably, there was no single journal that predominated among the top 25 cited references. Among the top three cited references were “Photothermal therapy and photoacoustic imaging via nano-theranostics in fighting cancer”, “Black Phosphorus Nanosheet-Based Drug Delivery System for Synergistic Photodynamic/Photothermal/Chemotherapy of Cancer”, and “Ultrasmall Black Phosphorus Quantum Dots: Synthesis and Use as Photothermal Agents”. However, upon closer examination, we discovered that these articles primarily offered a general overview of the relationship between 2D nano BP and cancer. To pinpoint the most significant citation bursts for 2D nano BP in cancer research, we employed CiteSpace with the following selection criteria: top 25, number of states: 2, and minimum duration: 2. This analysis identified 92 references exhibiting the strongest citation bursts, with 25 of them presented in Fig. 6. Among them, “Black phosphorus field-effect transistors (strength: 15.02),” “PEGylated WS2 Nanosheets as a Multifunctional Theranostic Agent for in vivo Dual-Modal CT/Photoacoustic Imaging Guided Photothermal Therapy (strength: 10.32),” and “High-Quality Black Phosphorus Atomic Layers by Liquid-Phase Exfoliation (strength: 9.63)” were the top three with the most vigorous citation bursts. Interestingly, the titles of the three most cutting-edge citation bursts were “Black phosphorus field-effect transistors”, “PEGylated WS2 Nanosheets as a Multifunctional Theranostic Agent for in vivo Dual-Modal CT/Photoacoustic Imaging Guided Photothermal Therapy” and “High-Quality Black Phosphorus Atomic Layers by Liquid-Phase Exfoliation”. To gain further insights into the research frontiers and hotspots in the field of 2D nano BP in cancer, we corresponded the DOIs of the 25 citations in Fig. 6 to the titles. These findings suggest that 2D nano BP has gained significant attention in recent years, particularly in the context of cancer therapy. It is worth noting that although 2D nano BP have potential application prospects in cancer therapy, however, there are still many obstacles to overcome from research and development to application.

**Table 8:** Top 25 cited references related to 2D nano BP in cancer

Paper	DOI	Total citations	TC per year
Liu Yj, 2019, Chem Soc Rev	10.1039/c8cs00618k	1619	323.8
Chen Ws, 2017, Adv Mater	10.1002/adma.201603864	916	130.86
Sun Zb, 2015, Angew Chem Int Edit	10.1002/anie.201506154	819	91
Wang H, 2015, J Am Chem Soc	10.1021/jacs.5b06025	774	86
Shao Jd, 2016, Nat Commun	10.1038/ncomms12967	760	95
Tao W, 2017, Adv Mater	10.1002/adma.201603276	657	93.86
Qiu M, 2018, P Natl Acad Sci Usa	10.1073/pnas.1714421115	583	97.17
Zeng Xw, 2018, Adv Sci	10.1002/advs.201800510	377	62.83
Chen Jm, 2020, Biomaterials	10.1016/j.biomaterials.2020.119827	367	91.75
Sun Cx, 2016, Biomaterials	10.1016/j.biomaterials.2016.03.022	351	43.88
Wang Xw, 2016, Chem Soc Rev	10.1039/c5cs00811e	345	43.13
Ji Xy, 2018, Adv Mater	10.1002/adma.201803031	321	53.5
Liu S, 2020, Angew Chem Int Edit	10.1002/anie.201911477	320	80
Tao W, 2017, Angew Chem Int Edit	10.1002/anie.201703657	310	44.29
Qiu M, 2018, Chem Soc Rev	10.1039/c8cs00342d	309	51.5
Chen Hb, 2018, Sci China Chem	10.1007/s11426-018–9397-5	304	50.67
Cheng L, 2020, Adv Mater	10.1002/adma.201902333	300	75
Liang X, 2019, J Control Release	10.1016/j.jconrel.2019.01.027	264	52.8
Yang Bw, 2018, Adv Mater	10.1002/adma.201705611	255	42.5
Shao Jd, 2018, Adv Sci	10.1002/advs.201700848	245	40.83
Sun Zb, 2017, Small	10.1002/smll.201602896	242	34.57
Yang D, 2017, Adv Funct Mater	10.1002/adfm.201700371	229	32.71
Choi Jr, 2018, Theranostics	10.7150/thno.22573	219	36.5
Xing Cy, 2018, Adv Healthc Mater	10.1002/adhm.201701510	212	35.33
Gui Rj, 2018, Chem Soc Rev	10.1039/c8cs00387d	205	34.17

### Keyword clusters and evolution

**Fig. 7:**
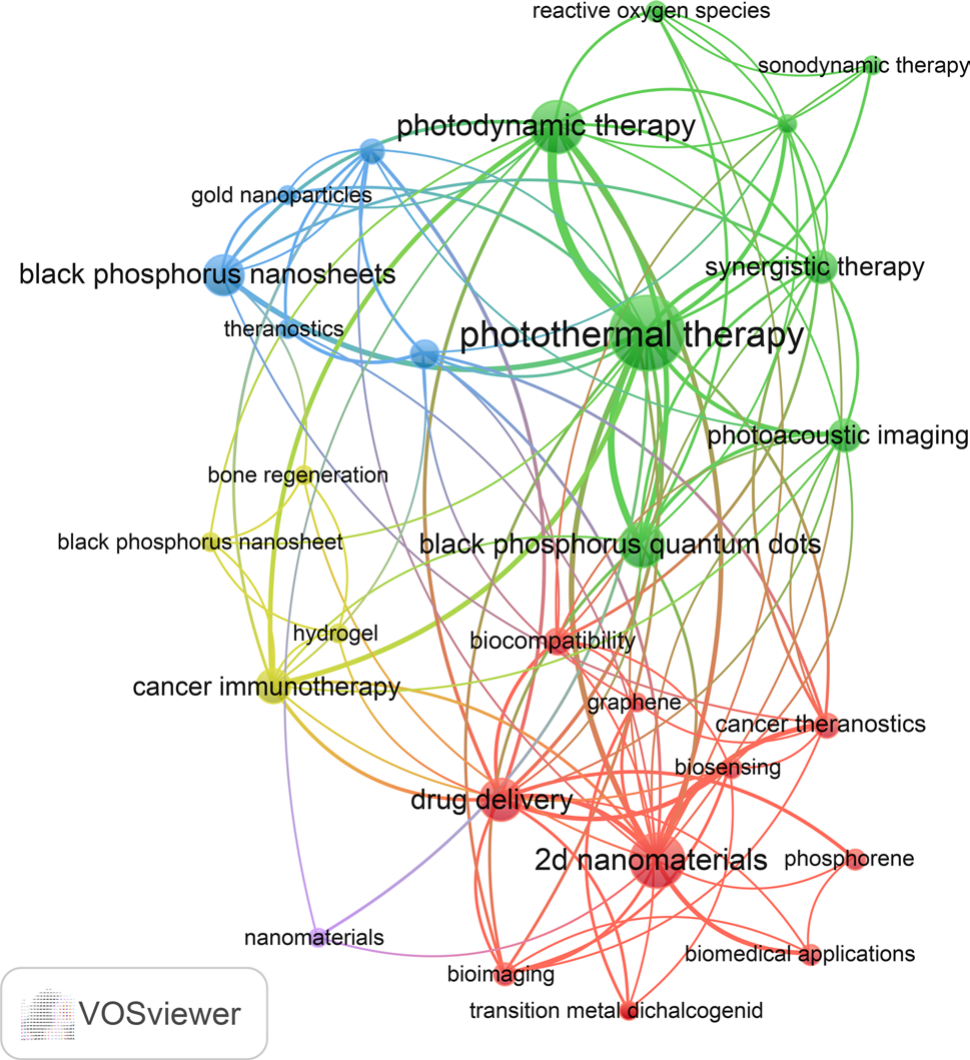
Keywords co-occurrence map of publications on 2D nano BP in cancer

**Fig. 8:**
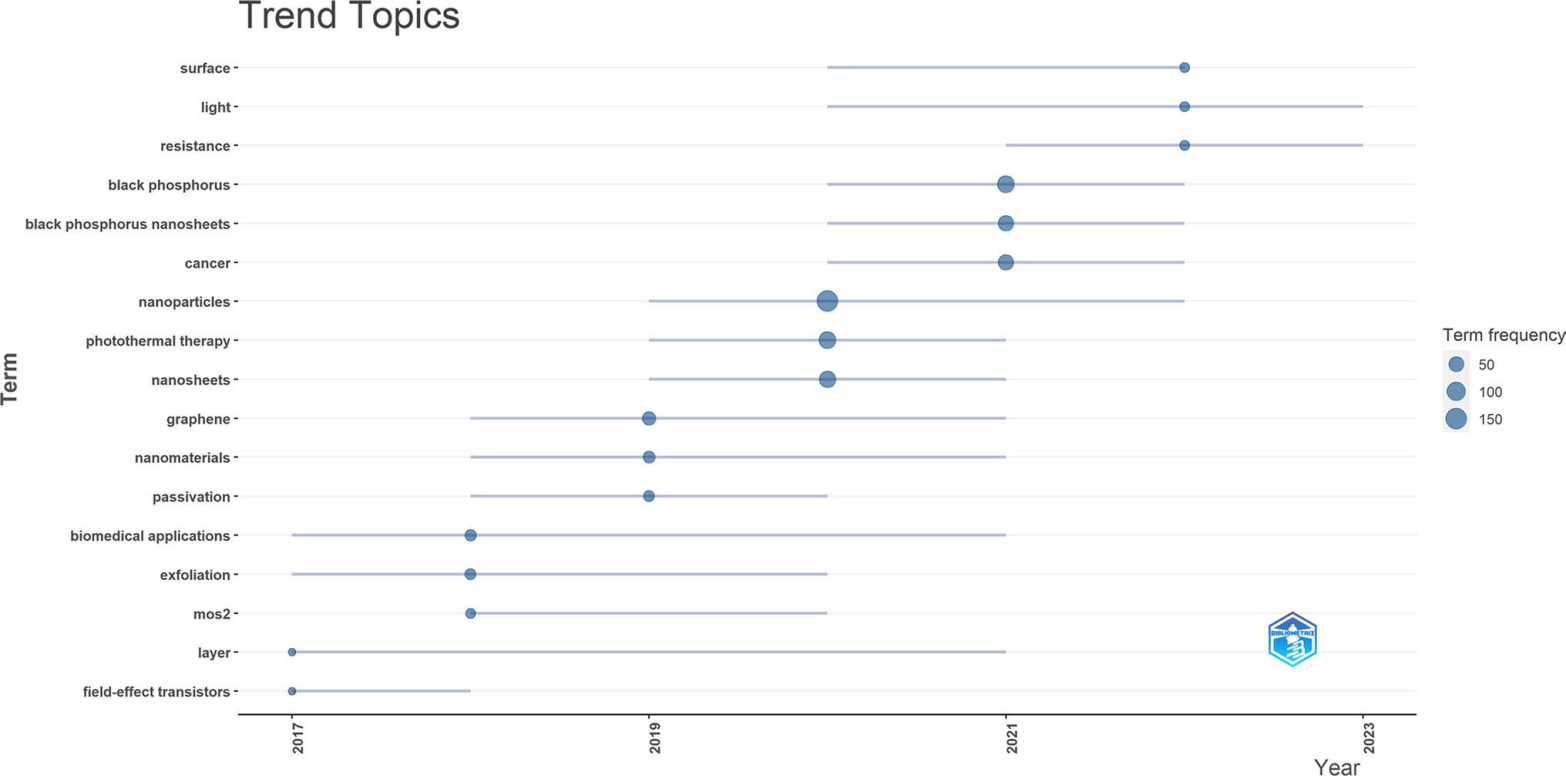
Trend topics on 2D nano BP in cancer research

Keyword clusters are an excellent way to understand the research hotspots and directions in a field. In this study, a total of 930 keywords were extracted using VOSviewer. Table 9 shows that the top 10 keywords appear more than 10 times. The most frequently occurring keywords were photothermal therapy (n = 84), followed by 2d nanomaterials (n = 51), photodynamic therapy (n = 43), black phosphorus quantum dots (n = 31), black phosphorus nanosheets (n = 30). Then, 26 keywords were selected according to the “minimum number of occurrences of a keyword ≥ 5” to draw the keyword cluster map (Fig. 7), and a total of five clusters with different colors are observed in Fig. 7. There were 10 keywords in the first cluster (red dot) with the theme of application of 2D nano BP in drug delivery, which including 2D nanomaterials, drug delivery, biocompatibility, bioimaging; biomedical applications. There were 8 keywords in the second cluster (green dot) with the theme of application of 2D nano BP quantum dots in PDT, which including black phosphorus quantum dots, multimodal imaging, photoacoustic imaging, PDT, PTT. There were 5 keywords in the third cluster (blue dot) with the theme of multiple applications of 2D nano BP nanosheets in cancer immunotherapy, which including black phosphorus nanosheets, gold nanoparticles, bone regeneration, cancer immunotherapy, hydrogel. There were 3 keywords in the fourth cluster (yellow dot) with the theme of integration of nanomedicine treatment and diagnosis, which including nanomedicine, nanoparticles, theranostics. There was 1 keyword in the fifth cluster (purple dot), nanomterials. In addition, we utilized the bibliometrix package of R software to generate a trend topic map **(**Fig. 8**)**. The trend topic map is a valuable tool for tracing the chronological development of specific research themes in a particular field. It allows us to analyze the evolution of that field over time and gain insights into its progression. By visually mapping the trends in Fig. 8, we were able to identify the research focus and evolution track of each stage of 2D nano BP in cancer research. Our findings indicate that current research in this field mainly focuses on 2D nano black phosphorus materials and the applications of black phosphorus in nanomedicine. In general, through keyword clusters and evolution, we found that the research hotspots of 2D nano BP in cancer mainly focus on the application prospects of nano 2D nano BP in PTT and PDT, the advantages of 2D nano BP drug carriers, and the application prospects of 2D nano BP in tumor immunotherapy. Among them, 2D nano BP drug carriers have unique advantages in the field of nano drug targeting carriers due to their better biocompatibility, larger surface area ratio, and modifiability compared to previous drug targeting carriers (such as gold nanoparticles).

**Table 9:** Top 10 keywords related to 2D nano BP on cancer

Rank	Keyword	Count
1	photothermal therapy	84
2	2d nanomaterials	51
3	photodynamic therapy	43
4	black phosphorus quantum dots	31
5	black phosphorus nanosheets	30
6	drug delivery	27
7	cancer immunotherapy	18
8	synergistic therapy	16
8	photoacoustic imaging	16
10	nanomedicine	12

## Discussion

### General information

To gain a deeper insight into the research focus and trajectory of 2D nano BP in cancer, we conducted a bibliometric analysis coupled with data visualization. Our study encompassed a total of 446 papers published between 2015 and 2023. The results revealed a progressive increase in the number of publications on 2D nano BP in cancer research over the last eight years, with a marked acceleration in the past five years. This trend indicates a growing interest and favorability among researchers towards 2D nano BP in the context of cancer. In the research field of 2D nano BP in cancer, China had published the most quantity of papers (n = 359). Furthermore, there are 8 affiliations in the top 10 affiliation publishing papers were from China, with Shenzhen University having the largest number of published papers (n = 64). The results are surprising, as China holds a leading position in the research of nano black phosphorus in the field of cancer treatment in the world. It is worth noting that Korea was the second country to publish papers in this field, followed by the India, indicating that Asian countries are involved in research in this field more than countries from other six continents and 2D nano BP in cancer has received worldwide attention from researchers. A total of 446 papers were published in 153 journals, with the leading journals including the Journal of Advanced Materials, Acs Nano, Angewandte Chemie-International Edition, Acs Applied Materials & Interfaces, and Biomaterials. Interestingly, the journal of Advanced Materials and Acs Nano were the most cited journals, and the journals of Acs Applied Materials & Interfaces and Biomaterials with the most public articles. This suggests that these journals are key publications in the field of 2D nano BP in cancer research. It is worth noting that the majority of authors who have published the highest number of documents and received the most citations within the top 10 are Chinese. These authors, including Zhang Han, Yu Xuefeng, Tao Wei, and Chu Paul K, have formed a research team that demonstrates China's leading position in this field. This observation highlights the significant contributions and expertise of Chinese researchers in the field of 2D nano BP in cancer research. At present, exhibiting significant potential for development. Further in-depth research is warranted to explore and advance our understanding of this area.

### Hotspots and development trends

By analyzing the most cited references, reference bursts, keyword clusters, and keyword trend topics, we identified the research hotspots and frontiers of 2D nano BP in cancer. Our study revealed three noteworthy aspects that deserve attention in this field.

The first research hotspot and development trend is the application of 2D nano BP as drug targeting carriers. Targeted therapy is a technique used to selectively deliver drugs to cancer cells or diseased sites with specific molecular targets, offering an available treatment option [44, 45]. Drug targeting vectors are tools designed to transport drugs and guide them to specific targets. They play a crucial role in enhancing drug selectivity, bioavailability, and efficacy, while minimizing harm to normal cells [46]. According to our keyword cluster analysis, "2D nanomaterials" and "drug delivery" hold the highest proportions. 2D nano BP is a novel genre of nanomaterial that can be applied in the field of cancer treatment, serves as a carrier for drug delivery and contributes to targeted therapy in the field of cancer treatment. Numerous studies have demonstrated that 2D nano BP, as an emerging targeting carrier, possesses several distinct advantages over conventional carriers. For instance, these studies [47, 48] have demonstrated that 2D nano BP exhibits superior biocompatibility compared to certain metallic nanoparticles (e.g., gold) and polymer-based nanoparticles. Notably, 2D nano BP's composition is more akin to natural biological materials, potentially mitigating toxicity issues within the body. In addition, the study [49] highlights that 2D nano BP possesses an extensive specific surface area, which enhances its capacity for drug adsorption, significantly exceeding that of conventional nanocarriers like nanoliposomes. This extensive surface area facilitates the effective loading and release of therapeutic agents. The selective permeability of the blood–brain barrier is critical for protecting the central nervous system by preventing the ingress of harmful substances [50]. But it has also become an obstacle for drugs to effectively enter the brain. This study indicates the preparation of non functionalized BP through esterification reaction between the carboxyl group of L-arginine and the hydroxyl group formed by preliminary oxidation on the surface of 2D nano BP. Then, glucose oxidase is combined with Arg through an amidation reaction to form a multimodal nanodrug with the ability to cross the blood–brain barrier [51]. It is important to highlight that several studies have shown the favorable biocompatibility of 2D nano BP. However, the metabolic pathway of 2D nano BP within the human body remains a subject of debate, and a definitive conclusion has yet to be reached. Consequently, further research is warranted in order to shed more light on this matter.

It is worth mentioning that the tumor microenvironment plays a key role in tumor survival, progression and metastasis [52]. Unfortunately, no studies have been conducted on 2D nano BP. Moreover, it is evident that the utilization of 2D nano BP in targeted therapy holds significant potential and represents a prominent research topic and trend within the field of cancer treatment. Continued investigations in this area are likely to yield valuable insights and advancements in the future.

The second research hotspot and development trend involves the application of 2D nano BP in PTT and PDT. PTT involves the injection of photosensitizers, such as metal nanoparticles, into the body or their local application via light. This process presents a promising alternative for tumor suppression by activating autophagy or inhibiting cell signaling pathways that influence the cell cycle, ultimately inducing cell death [53–55]. Meanwhile, PDT is a method that uses the energy emitted by a laser source with a specific wavelength to activate photosensitizers, causing them to release reactive oxygen species or other photochemical substances, and generating cytotoxic reactions to kill cancer cells for therapeutic purposes. [56]. Our keyword cluster analysis shows that "photothermal therapy", "photodynamic therapy" and "black phosphorus quantum dots" occupy the top three places in the cluster. The results show that 2D nano BP plays the role of photosensitizer, which can convert light energy into heat and oxidizing substances respectively, and participates in the PTT and PDT in the field of cancer treatment. In fact, many studies have shown that the combination of 2D nano BP as a new photosensitizer with traditional photosensitizers can break through the limitations of many traditional photosensitizers. The studies [57, 58] indicates that 2D nano BP has high photothermal conversion efficiency and absorption ability, especially for near-infrared(NIR), which can more effectively absorb light energy and generate local heating. Moreover, 2D nano BP has a wider spectral absorption range. Using 2D nano BP as a photosensitizer for PTT can expand the scope of deep tissue therapy. The study [59] show that by using polyamides and hyaluronic acid layers to effectively modify and functionalize the surface of 2D nano BP tablets, doxorubicin can be loaded to achieve the photothermal killing target of specific tumor cells, improve the accuracy of PTT and reduce the impact on healthy tissues. The study [58] shows that 2D nano BP is a controllable material, which can be used to form a 3D network with irregular micron pores and thin and strong cellulose forming walls by combining the sheet hydrogels of nano black scales with cellulose, showing good biological stability and flexibility in the control of material procedures. Through these studies, we can find that the research hotspots of 2D nano BP in the field of targeted therapy focus on "biocompatibility", "large load" and "through the blood–brain barrier". Indeed, the shape, size, and surface characteristics of 2D nano BP can be modified or combined with other materials to alter its photothermal properties. This flexibility opens up possibilities for selecting and designing suitable 2D nano BP photosensitizers that can cater to different medical conditions. It is important to note that while 2D nano BP exhibits high biocompatibility, it is often combined with other materials to leverage their strengths and mitigate weaknesses, thus serving as a targeted photosensitizer. Undoubtedly, the application of 2D nano BP in PTT and PDT remains a prominent and evolving research topic in the field of cancer treatment. Its potential and trend in this area further underscore its significance.

The third hotspot and trend pertains to the application of 2D nano BP in immunotherapy. Immunotherapy is a treatment modality focused on enhancing the body’s immune system to effectively combat diseases. It aims to bolster, regulate, or restore the immune function in order to impede disease progression and target specific ailments. Our keyword cluster analysis reveals that "black phosphorus nanosheets" and "cancer immunotherapy" hold the top two positions within the cluster, illustrating the significant role played by 2D nano BP in the realm of immunotherapy. Indeed, several studies have demonstrated the role of 2D nano BP as an immune adjuvant, which enhances the sensitivity of the immune system and improves the efficacy of immunotherapy. This contribution positions 2D nano BP as a valuable component within the realm of cancer treatment, specifically in the field of immunotherapy. For example, this study [60] shows that integrating heme onto the surface of 2D nano BP nanosheets enhances the efficiency of endogenous immune activation behavior, amplifies the inflammatory response and activates the anti-tumor effect of the immune system. At the same time, this study [61] obtained HA hyaluronic acid (HA)-BP by targeting multifunctional BP nanoparticles modified with polyethylene glycol HA. It was found that it downregulated the expression of CD206 (M2 macrophage marker) by 42.3%, and upregulated the proportion of CD86 (M1 macrophage marker) by 59.6%, indicating that HABP nanoparticles have the function of reshaping the tumor associated macrophage (TAM) phenotype (from pre tumor M2 TAM to anti-tumor M1 TAM). In addition, this study [62] shows that the 2D nano BP doped with N-isopropylacrylamide hydrogel particles forms hydrogel particles with interconnected nanopores. This can stimulate the proliferation and differentiation of γδT cells to resist the growth of tumor cells, thereby providing direct anti-tumor activity and enhancing the ability of γδT cells to attack tumor cells. These studies demonstrate that 2D nano BP can serve as an immune enhancer to enhance the function of related immune cells and achieve the goal of inhibiting tumor cell growth. These studies indicate that 2D nano BP can be combined with other materials to form immune enhancers to enhance the function of related immune cells to inhibit the growth of tumor cells. Notably, in the immunotherapy involving 2D nano BP, the treatment mode is often not single, and in most cases it will form a combined therapy system with targeted therapy, PTT, PDT, etc. Therefore, it is evident that the application of 2D nano BP in immunotherapy has become a hot topic and trend in the field of cancer treatment research.

In summary, based on the above analysis, future research in this field should focus on optimizing the preparation methods of nano black phosphorus to enhance its stability and biocompatibility. Additionally, integrating nano black phosphorus into multifunctional therapeutic platforms for synergistic therapy and designing targeted drug delivery systems can improve therapeutic efficacy and reduce side effects.

### Strengths and limitations

This research can provide valuable insights for researchers seeking a better understanding of this field and exploring new directions. However, it is crucial to acknowledge certain limitations. Firstly, our study relied solely on the WoSCC database as the source of data, which may have led to the exclusion of some publications. Nevertheless, it is important to note that the WoS database is highly regarded by researchers and widely recognized as a high-quality digital literature database, making it a suitable choice for bibliometric analysis [63, 64]. Therefore, the data source we had chosen with high credibility. Secondly, we only analyzed publications in English, it may cause source bias. Despite these limitations, our research offers a comprehensive overview of the overall landscape, key areas of interest, and research trends within this field. By taking into account these factors, readers can gain valuable insights into the general state of the field and identify potential avenues for further exploration.

## Conclusions

Our research clearly demonstrates the main research hotspots and frontiers of 2D nano BP in cancer treatment research. The following is a summary of the knowledge points and research hotspots in the field of 2D black phosphorus anti-cancer:

- 2D nano BP has attracted researchers worldwide in cancer research, with Korea, India, the United States, and Iran being the most active countries. With the deeper of research on 2D nano BP anticancer in the future, and cooperation between countries will continue to be closer.
- The Journal of ACS Applied Materials & Interfaces was the most published and The Journal of Advanced Materials was the most cited journal on 2D nano BP in cancer related literature. The journal of ACS Applied Materials & Interfaces and the journal of Advanced Materials are representative journals in the field of 2D nano BP research.
- The co-authors of 2D nano BP reveals extensive and close collaboration among authors, with Zhang Han, Tao Wei, and Yu Xuefeng serving as representative centers of collaboration.
- The application of 2D nano BP in targeted therapy has become a hot topic and trend in the field of cancer treatment research.
- The application of 2D nano BP in PTT and PDT has emerged as a hotspot and trend in cancer treatment research.
- The application of 2D nano BP in immunotherapy has become a hot topic and trend in the field of cancer treatment research.

In conclusion, our study offers valuable insights into the trends and hotspots of 2D nano BP in cancer treatment research. These findings can swiftly aid researchers in comprehending the field and enable the exploration of new avenues for future investigations. By identifying current research limitations and potential areas of focus, our study equips researchers with valuable information to effectively delve into this area and encourages them to pursue innovative directions in their research.

## Data Availability

Data will be made available on request. Data from web of science database. Available through the following URL: https://www.webofscience.com

## Abbreviations

2D nano BP: Two-Dimensional Nano Black Phosphorus
IFs: The journal impact factors
JCR: Journal Citation Reports
MCPs: Multiple country publications
NIR: Near-infrared
PDT: Photodynamic therapy
PTT: Photothermal therapy
TAM: Tumor associated macrophage
WOSCC: Web of Science core data collection
